# External Exposome Factors and Adverse Heart Failure Outcomes in the OneFlorida+ Network: Retrospective Cohort Study

**DOI:** 10.2196/71595

**Published:** 2025-08-25

**Authors:** Wenxi Huang, Stephen E Kimmel, Mustafa Ahmed, Steven M Smith, Yao An Lee, Lanting Yang, Inmaculada Hernandez, Jiang Bian, Jingchuan Guo

**Affiliations:** 1 Department of Pharmaceutical Outcomes and Policy, College of Pharmacy, University of Florida, 1889 Museum Road, DSIT 6004, Gainesville, FL, 32606, United States, 1 3522736533; 2 Department of Epidemiology, College of Public Health and Health Professions and College of Medicine, University of Florida, Gainesville, FL, United States; 3 Department of Medicine, College of Medicine, University of Florida, Gainesville, FL, United States; 4 Division of Clinical Pharmacy, Skaggs School of Pharmacy and Pharmaceutical Sciences, University of California, San Diego, La Jolla, CA, United States; 5 Department of Biostatistics and Health Data Science, School of Medicine, Indiana University, Indianapolis, IN, United States; 6 Center for Biomedical Informatics, Regenstrief Institute, Indianapolis, IN, United States; 7 Department of Medicine, Melvin and Bren Simon Comprehensive Cancer Center, Indiana University, Indianapolis, IN, United States; 8 Indiana University Health, Indianapolis, IN, United States

**Keywords:** machine learning, social determinants of health, heart failure, electronic health record, climate change

## Abstract

**Background:**

Heart failure (HF) readmission rates vary across geographic regions in the United States, yet the impact of external exposome factors, such as contextual-level social determinants of health (SDoH), on adverse HF outcomes is not well understood.

**Objective:**

This study aims to examine the association between external exposome factors and the risk of HF readmission and all-cause mortality using a data-driven approach.

**Methods:**

We conducted a retrospective cohort study using electronic health record (EHR) data from the OneFlorida+ Network, including patients hospitalized for HF (HHF) from 2016 to 2022. A total of 1308 external exposome factors, covering a wide range of SDoH (eg, economic stability, education, health care access, natural and built environments, and social context), were linked to patients’ EHR data based on their county-level residential location. Patients were followed for 1 year after their first HHF to capture readmission and mortality events. We applied the least absolute shrinkage and selection operator regularization to preselect candidate variables, followed by a 2-phase external exposome-wide association study using mixed-effects logistic regression to identify key factors associated with the composite outcome of 1-year HF readmission and mortality.

**Results:**

Among 63,940 patients with HF (n=30,475, 48% women; mean age 65, SD 14 years), higher maximum temperature in May was significantly associated with increased risk of the composite outcome (adjusted odds ratio [aOR] 1.04, 95% CI 1.02-1.06; *P*<.001). Subgroup analyses showed consistent associations across age, sex, race, socioeconomic status, and rural or urban strata.

**Conclusions:**

Using a data-driven approach, we found that elevated maximum temperature in May (late spring) was significantly associated with HF readmission and mortality in Florida. Further investigations are warranted to uncover the intricate mechanisms through which extreme heat potentially influences HF outcomes.

## Introduction

### Background

Heart failure (HF) is a significant public health challenge in the United States, being the second most common reason for hospital admissions. It accounts for over 4.4 million cases leading to hospitalization annually, with health care costs surpassing US $31 billion [1]. Despite advances in treatment and management strategies, HF remains a chronic and progressive condition with limited prospects for a cure [2]. For patients hospitalized with HF, readmission rates and mortality serve as key quality indicators, reflecting the efficacy of both inpatient treatment and postdischarge care [3,4]. Approximately 55% of adult patients with HF are readmitted within one year, and the one-year mortality rate is about 25% [5-7].

Large geographic variation exists in the prevalence of HF and the incidence of complications [8], suggesting that the environment may play a critical role in the disease course. Extensive research has delineated clinical predictors of HF hospitalization trajectories; however, less clearly defined is the role of contextual-level socioeconomic, built, and natural environments as modifiable upstream factors impacting patients’ hospitalization patterns and mortality risk [9-13]. Such contextual factors are increasingly recognized to influence health outcomes and serve as a critical source of information to develop policy interventions designed to improve population health management and value-based care [14,15]. While previous studies have identified multiple contextual exposures associated with HF outcomes in the natural, built, and social environments [9-13], these studies only focused on a subset of preselected environmental factors. In addition, these studies assessed these factors in isolation without considering the totality of the environment or the external exposome [16]. Significant knowledge gaps remain regarding the combined effects of multiple environmental exposures on cardiovascular health, and there is a need for a more comprehensive approach that considers the full spectrum of external factors an individual is exposed to over a lifetime.

The external exposome-wide association study (ExWAS) framework addresses this need by providing a systematic and efficient approach to screen hundreds of environmental exposures simultaneously, offering an opportunity to identify novel environmental factors associated with HF outcomes [17,18].

### Objective

In this study, we applied the ExWAS framework to identify novel external exposome factors associated with HF readmission and mortality [16,18-20]. We used electronic health records (EHRs) to examine the association between the external exposome and the risk of HF admission and mortality. Specifically, we integrated data on a wide range of external exposome factors, including economic stability, access to and quality of education, health care access, the natural environment, and the neighborhood and built environment surrounding patients who were hospitalized for heart failure (HHF). A more nuanced understanding of which external exposome factors influence adverse HF outcomes is critical to tailor care coordination, transitional support, and preventive services to patients, ultimately helping clinicians and health care systems reduce the HF burden.

## Methods

### Data Source and Study Population

We conducted a retrospective cohort study using 2016‐2022 EHRs from the OneFlorida+ Clinical Research Network [21]. OneFlorida+ Clinical Research Network is one of the largest statewide clinical data repositories, encompassing data from 13 unique health care systems that collectively serve approximately half of Florida’s population (~17.2 million individuals), covering all 67 Florida counties [22]. OneFlorida+ data are racially and ethnically diverse, with a mixture of rural-urban populations, reflecting national demographic changes, thus enhancing the generalizability of our study [22,23]. In addition, OneFlorida+ is linked with the National Death Index, allowing for mortality status tracking of its patients [22]. These longitudinal, patient-level data were chosen to enable a comprehensive analysis of HF adverse outcomes by leveraging extensive clinical and demographic information across a large, diverse population in Florida.

The study cohort included patients aged ≥18 years who had at least one HHF between January 1, 2016, and January 31, 2021. We excluded individuals missing a residential history (county), patients residing outside of Florida, and those with less than one year of health care encounter history before the first observed HHF. HHF was defined as a primary admission diagnosis with HF, identified using *ICD-10* (*International Classification of Diseases, Tenth Revision*) codes (I50.x, I11.0, I13.0, I13.2, I97.13, and I09.81) [24]. This algorithm was previously validated against discharge summary or medical record by Thygesen et al [24], with a positive predictive value of 100% (95% CI 92.9‐100) based on primary diagnoses.

The index date was defined as the date of the patient’s first HHF recorded in the EHR during the study period. We followed each patient for one year after their index hospitalization until death or the end of the study period, whichever occurred first.

### Study Outcome

Our primary outcome was a composite of HF readmission or all-cause mortality within one year after the index date, defined as HF readmission or death, whichever occurred first. We included death as part of the composite outcome to account for competing risks. In HF studies, patients who die before a potential readmission are no longer at risk for readmission, which could bias readmission risk estimates. By incorporating death into the composite outcome, we provide a more accurate estimate of the true burden of HF, capturing both readmission and mortality risks. HF readmission was identified using *International Classification of Diseases, Ninth Revision: Clinical Modification* (*ICD-9-CM*) and *International Classification of Diseases, Tenth Revision: Clinical Modification* (*ICD-10-CM*) codes as mentioned in the “Data Source and Study Population” section. All-cause mortality was identified by death records in OneFlorida+ EHR, which was linked with the National Death Index. Our secondary outcome was HF readmission alone within one year after the index date.

### Exposures of Interest

Our exposures of interest included 1308 contextual-level exposome factors obtained from 11 well-validated sources, including the Agency for Healthcare Research and Quality [25], County Health Roadmap [26], Local Area Unemployment Statistics [27], economic resilience [28], US Cancer Statistics [29], Dartmouth Health [30], Air Quality Index [31], Air Quality System [31], Local Area Unemployment Statistics [27], religion [32], water [33], and social capital [34]. We categorized these factors into 6 domains, inspired by the Healthy People 2030 framework [35] (refer to Table 1) social and community context (eg, food access), neighborhood and built environment (eg, hospital density), natural environment (eg, temperature), health care access and quality (eg, insurance), education access and quality (eg, graduation rate), and economic stability (eg, income). These contextual-level exposome factors were temporospatially linked to each patient’s EHR data based on their residential histories at the county level, using Federal Information Processing Standard codes.

**Table 1.** Summary of external exposome measures included in our retrospective cohort, exposome‑wide analysis.

Category and data source^a^	Time period	Number of variables
Economic stability		
Agency for Healthcare Research and Quality	2009‐2019	88
County Health Roadmap	2009‐2019	10
Local Area Unemployment Statistics	2019	4
Economic resilience	2015	3
Education access and quality		
Agency for Healthcare Research and Quality	2009‐2019	19
County Health Roadmap	2009‐2019	2
Health care access and quality		
US Cancer Statistics	2018	8
Agency for Healthcare Research and Quality	2009‐2019	451
County Health Roadmap	2009‐2019	54
Dartmouth Health	2009‐2019	8
Natural environment		
Agency for Healthcare Research and Quality	2009‐2019	97
Neighborhood and built environment		
Agency for Healthcare Research and Quality	2009‐2019	117
Air Quality Index	2009‐2019	15
Air Quality System	2009‐2019	14
County Health Roadmap	2009‐2019	18
Local Area Unemployment Statistics	2009‐2019	2
Religion	2010	3
Water	2017	242
Social and community context		
Social capital	2009‐2014	13
Agency for Healthcare Research and Quality	2009‐2019	138
County Health Roadmap	2009‐2019	2

a All data sources are spatially scaled at the county level.

### Covariates

Covariates were measured during the lookback period (1 year before the index HHF). Covariates were selected based on previous studies [36-39] and clinical experience. These included age, race/ethnicity (non-Hispanic White, non-Hispanic Black, Hispanic, and others), sex, HF subtype (HF with reduced vs preserved ejection fraction), and comorbidities (chronic obstructive pulmonary disease [COPD], myocardial infarction, anemia, diabetes, cancer, and the use of an implantable cardioverter-defibrillator [ICD], cardiac resynchronization therapy [CRT], or both), as well as medication use (sacubitril/valsartan, sodium-glucose cotransporter-2 inhibitors, nitrates [isosorbide mononitrate, nitroglycerin, isosorbide dinitrate], loop diuretics, and angiotensin-converting enzyme inhibitors). Comorbidities were identified using *ICD-9-CM* or *ICD-10-CM* diagnosis and procedure codes (Table S1 in Multimedia Appendix 1) [9,40-43]. Medication use was identified from patient prescription records.

### Area Deprivation Index

The Area Deprivation Index (ADI) is a metric developed to assess the level of socioeconomic disadvantage in a given geographic area [44]. It was initially created by researchers at the University of Wisconsin-Madison and has become widely used in health research to understand how neighborhood-level socioeconomic factors influence health outcomes. The ADI provides a composite score based on several indicators, capturing various dimensions of deprivation, such as education, employment, housing quality, and income [44]. The ADI is a continuous value, with higher scores indicating areas with greater socioeconomic disadvantage. We converted it to a dichotomized variable, with the top 50% ADI defined as high deprivation. Each patient’s ADI was linked at the patient level, based on their Federal Information Processing Standard code.

### Statistical Analysis

In the initial stage of our analysis, we conducted data preprocessing for the contextual-level external exposome factors (Figure S1 in Multimedia Appendix 1). We performed normalization transformations on all continuous variables using the bestNormalize package [45] in R statistical software (version 3.6.1; R Development Core Team), which supports various transformation methods, including logarithmic, square root, exponential, arcsinh, Box-Cox, and Yeo-Johnson transformations. The optimal transformation method was selected based on the Pearson *P* statistics. Next, we examined the correlation among variables and removed highly correlated predictors (correlation >0.9) to reduce redundancy. We then used the least absolute shrinkage and selection operator (LASSO) for variable selection to identify key contextual-level variables [46]. The LASSO model was fit using the glmnet package [47], with cross-validation to determine the optimal regularization parameter (λ). To retain more variables, we used Elastic Net regularization, combining Ridge and LASSO penalties (α=.01). Cross-validation identified λ.min, the lambda value that yielded the minimum cross-validated error, which was then used to refit the model. We retained variables with nonzero coefficients in the final model, indicating significant predictors identified by LASSO. This approach effectively reduced multicollinearity while preserving model interpretability. The final set of selected variables was stored for subsequent analysis. Details of the selected transformations and parameters for preselected external exposome variables are provided in Table S2 in Multimedia Appendix 1. Furthermore, we standardized all continuous variables to a *z* score (mean 0, SD 1). The missingness rate for all contextual-level exposome factors and covariates of interest in our model was restricted to <50% [48]. Variables with >50% missingness were excluded for further analysis to avoid biased results and maintain reliability, as extensive missing data can limit the effectiveness of imputation and compromise study validity [49-52]. We imputed missing data for all external exposome factors and covariates using either the median or mode value, depending on the variable type [53].

We then used a 2-phase ExWAS approach, as presented in Figure S1 in Multimedia Appendix 1, to identify key contextual-level factors associated with 1-year HF readmission or mortality [20,54]. In phase 1, we randomly divided the entire dataset into discovery (50%) and replication (50%) sets. We then tested the association of each external exposome factor with our composite outcome using mixed-effects logistic regression models, adjusting for covariates in both sets. Mixed-effect models were fitted for each external exposome factor, adjusting for all potential confounders (age, race, sex, history of heart failure with preserved ejection fraction (HFpEF), myocardial infarction, COPD, anemia, diabetes, cancer, and the presence of ICD and/or CRT-D; prescription history of sacubitril/valsartan, sodium-glucose cotransporter-2 inhibitors, nitrates, loop diuretics, and angiotensin-converting enzyme inhibitors), with population size set as the offset and a random intercept by county. To address the issue of multiple testing, we applied the Benjamini-Hochberg procedure to restrict the false discovery rate at a 5% level [55]. Variables with false discovery rate–adjusted *P* values (or q values) <.05 in both the discovery and replication sets were deemed significant in phase 1. We then assessed the pairwise Pearson correlations among these variables to further reduce collinearity. For variable pairs with absolute correlation coefficients exceeding 0.7, we retained the variable with the lower mean absolute correlation across all variables [56]. In phase 2, we constructed a mixed-effects logistic regression model incorporating all remaining significant variables identified in phase 1, along with all covariates of interest, including age, race, sex, comorbidities, and medication history. We reported the results of this model using adjusted odds ratios (aORs) with corresponding 95% CI for variables that remained significant (q<0.05) in phase 2.

All analyses were conducted using the R statistical software (version 3.6.1; R Development Core Team). Subgroup analyses were performed based on race (ie, White, Black, and so on), sex (female and male), age (≥65 and <65 years), residential region (rural and urban), ADI level (low deprivation and high deprivation), and COVID-19 period (prepandemic: January 2016 to February 2020; pandemic: March 2020 to January 2021).

### Ethical Considerations

We obtained ethics approval from the University of Florida Institutional Review Board (202201080) before abstracting data. This study used retrospective, deidentified patient EHR data from the OneFlorida+ Network. Informed consent was waived by the review boards due to the retrospective nature of this research. No patients were directly recruited, and no individual compensation was provided. All data used complied with relevant patient privacy regulations, and no identifiable individuals are depicted in any figures or other study materials.

## Results

**Figure 1.**
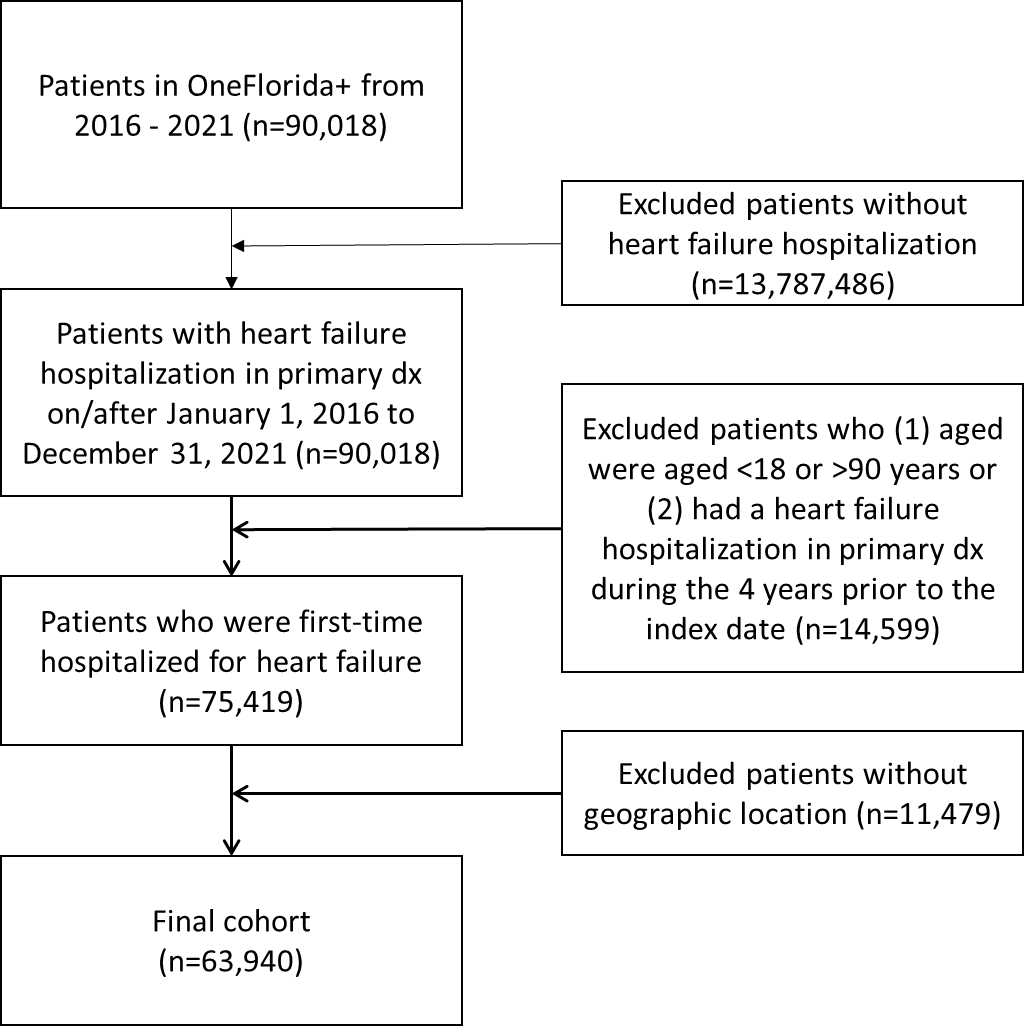
Patient flowchart. dx: diagnosis.

This study is reported following the STROBE (Strengthening the Reporting of Observational Studies in Epidemiology) guidelines for cohort studies. Our study cohort consisted of a total of 63,937 patients with an index HHF, of whom 30,475 (47.7%) were women, with a mean age of 65 (SD 14) years (Figure 1, Table 2, Checklist 1).

**Table 2.** Patient characteristics, by race and ethnicity, among patients hospitalized with heart failure in the OneFlorida+ Network.

Variable	Overall (n=63,940)^a^	Hispanic (n=12,380)^a^	NHB^b^ (n=17,853)^a^	NHW^c^ (n=27,484)^a^	Other (n=6223)^a^	*P* value^d^
Age (years), mean (SD)	65 (14)	68 (15)	61 (15)	68 (13)	65 (14)	<.001
Female, n (%)	30,475 (47.7)	5872 (47.4)	9024 (50.5)	12,766 (46.4)	2813 (45.2)	<.001
OneFlorida+ Network, n (%)						<.001
A	22,816 (35.7)	3535 (28.6)	5139 (28.8)	9834 (35.8)	4308 (69.2)	
B	11,558 (18.1)	2811 (22.7)	2353 (13.2)	5599 (20.4)	795 (12.8)	
C	10,119 (15.8)	387 (3.1)	3851 (21.6)	5538 (20.2)	343 (5.5)	
D	7807 (12.2)	3942 (31.9)	2710 (15.2)	1036 (3.8)	119 (1.9)	
E	6078 (9.5)	941 (7.6)	1791 (10.0)	3092 (11.2)	254 (4.1)	
Other	5562 (8.7)	764 (6.2)	2009 (11.2)	2385 (8.7)	404 (6.5)	
Anemia, n (%)	30,547 (47.8)	6169 (49.8)	9212 (51.6)	12,505 (45.5)	2661 (42.8)	<.001
COPD^e^, n (%)	23,309 (36.4)	4269 (34.5)	5636 (31.6)	11,395 (41.5)	2009 (32.3)	<.001
Diabetes, n (%)	32,615 (51.0)	7194 (58.1)	9655 (54.1)	12,817 (46.6)	2949 (47.4)	<.001
HFpEF^f^, n (%)	33,407 (52.2)	6424 (51.9)	9128 (51.1)	14,695 (53.5)	3160 (50.7)	<.001
History of myocardial infarction, n (%)	10,356 (16.2)	2070 (16.7)	2494 (14.0)	4831 (17.6)	961 (15.4)	<.001
ICD and/or CRT-D^g^, n (%)	5114 (8.0)	1072 (8.7)	1397 (7.8)	2228 (8.1)	417 (6.7)	<.001
Mechanical ventilation, n (%)	5863 (9.2)	1533 (12.4)	1911 (10.7)	1917 (7.0)	502 (8.1)	<.001
Cancer, n (%)	7419 (11.6)	1472 (11.9)	1859 (10.4)	3518 (12.8)	570 (9.2)	<.001
HF^h^ readmission, n (%)	21,442 (33.5)	4418 (35.7)	6847 (38.3)	8194 (29.8)	1983 (31.9)	<.001
HF readmission or all-cause mortality, n (%)	25,078 (39.2)	4977 (40.2)	7897 (44.2)	10,063 (36.6)	2141 (34.4)	<.001

a Values are means (SDs) for continuous variables and counts (%) for categorical variables. Values of polytomous variables may not sum to 100% due to rounding.

b NHB: non-Hispanic Black.

c NHW: non-Hispanic White.

d *P* values are based on the Kruskal-Wallis rank sum test or Pearson chi-square test, as appropriate.

e COPD: chronic obstructive pulmonary disease.

f HFpEF: heart failure with preserved ejection fraction.

g ICD and/or CRT-D: implantable cardioverter defibrillator and/or cardiac resynchronization therapy defibrillator within 4 years before the index date.

h HF: heart failure.

A total of 33,407 out of the study cohort (52.2%) had HFpEF. Patient characteristics by race and ethnicity are detailed in Table 2. Notably, non-Hispanic Black patients were younger and more likely to have diabetes, anemia, and require mechanical ventilation compared to non-Hispanic White patients. During the one-year follow-up, 33.5% of patients were readmitted for heart failure, and an additional 5.7% died without a prior heart failure readmission, resulting in 39.2% experiencing either heart failure readmission or all-cause mortality. In other words, patients who were readmitted did not overlap with those who died within the follow-up period, indicating that each patient contributed to only one outcome category. After data preprocessing and LASSO variable selection, 96 of the initial 1308 external exposome factors were retained for the ExWAS analysis (refer to Table S2 in Multimedia Appendix 1). In phase 1 of the ExWAS analysis, we identified 3 variables (median income of grandparent householder and/or spouse responsible for grandchildren younger than 18 years, percentage of households not receiving food stamps/Supplemental Nutrition Assistance Program (SNAP) with income below the poverty level, and maximum temperature in May) that were significantly associated with the composite outcome of HHF and death in both the discovery and replication sets (Table 3).

**Table 3.** Results from the external exposome-wide association study (ExWAS) in phase 1.

Exposure variable	Category	Transformation	Phase 1, discovery set		Phase 1, replication set		Phase 2	
			OR^a^ (95% CI)	q-value	OR^a^ (95% CI)	q-value	OR^a^ (95% CI)	q-value
Monthly (May) maximum temperature (°F)	Neighborhood and built environment	No transform	1.06 (1.03-1.10)	2.94E-03	1.04 (1.02-1.06)	3.67E-05	1.04 (1.02-1.06)	6.57E-05
Median income of grandparent householder and/or spouse responsible for grandchildren <18 years (US dollars, inflation-adjusted to data file year)	Economic stability	sqrt_x	0.95 (0.92-0.98)	3.86E-02	0.96 (0.94-0.98)	5.42E-03	0.98 (0.96-1.00)	7.64E-02
Percentage of households not receiving food stamps/SNAP^b^ with income below the poverty level	Economic stability	Yeo-Johnson	1.05 (1.02-1.07)	4.77E-02	1.05 (1.03-1.07)	3.67E-05	1.02 (1.00-1.04)	8.84E-02

a OR: odds ratio.

b SNAP: Supplemental Nutrition Assistance Program.

A visual summary of the phase 1 results is presented in Figure S3 in Multimedia Appendix 1 using a volcano plot. This figure illustrates the significance and aOR of the tested variables, with a solid dot indicating the variables that met the significance threshold in both sets. Some categories had zero significant factors, indicating no statistical significance for those exposures in both sets. To address the collinearity among the identified temperature-related variables, we calculated the mean absolute correlation between each pair of variables (Figure S4 in Multimedia Appendix 1).

In phase 2, all significant variables from phase 1 (median income of grandparent householder and/or spouse responsible for grandchildren younger than 18 years, percentage of households not receiving food stamps/SNAP with income below the poverty level, and maximum temperature in May) were incorporated into a multivariable mixed-effect binomial regression model, adjusting for all covariates (age, race, sex, history of HFpEF, myocardial infarction, COPD, anemia, diabetes, cancer, and the presence of ICD and/or CRT-D). Only the maximum temperature recorded in May for the county where the patient resides maintained its statistical significance in the phase 2 analysis, with an aOR of 1.04 (95% CI 1.02-1.06) (Table 3).

Although we only retained the May maximum temperature in the final model, we found that some other monthly temperature variables were also associated with adverse HF outcomes (Table S3 in Multimedia Appendix 1). Our final model retained a single temperature variable based on LASSO regularization, which prioritized predictive strength while mitigating multicollinearity. As such, the broader dynamics of temperature patterns and cumulative heat exposure warrant further exploration in future studies.

**Figure 2.**
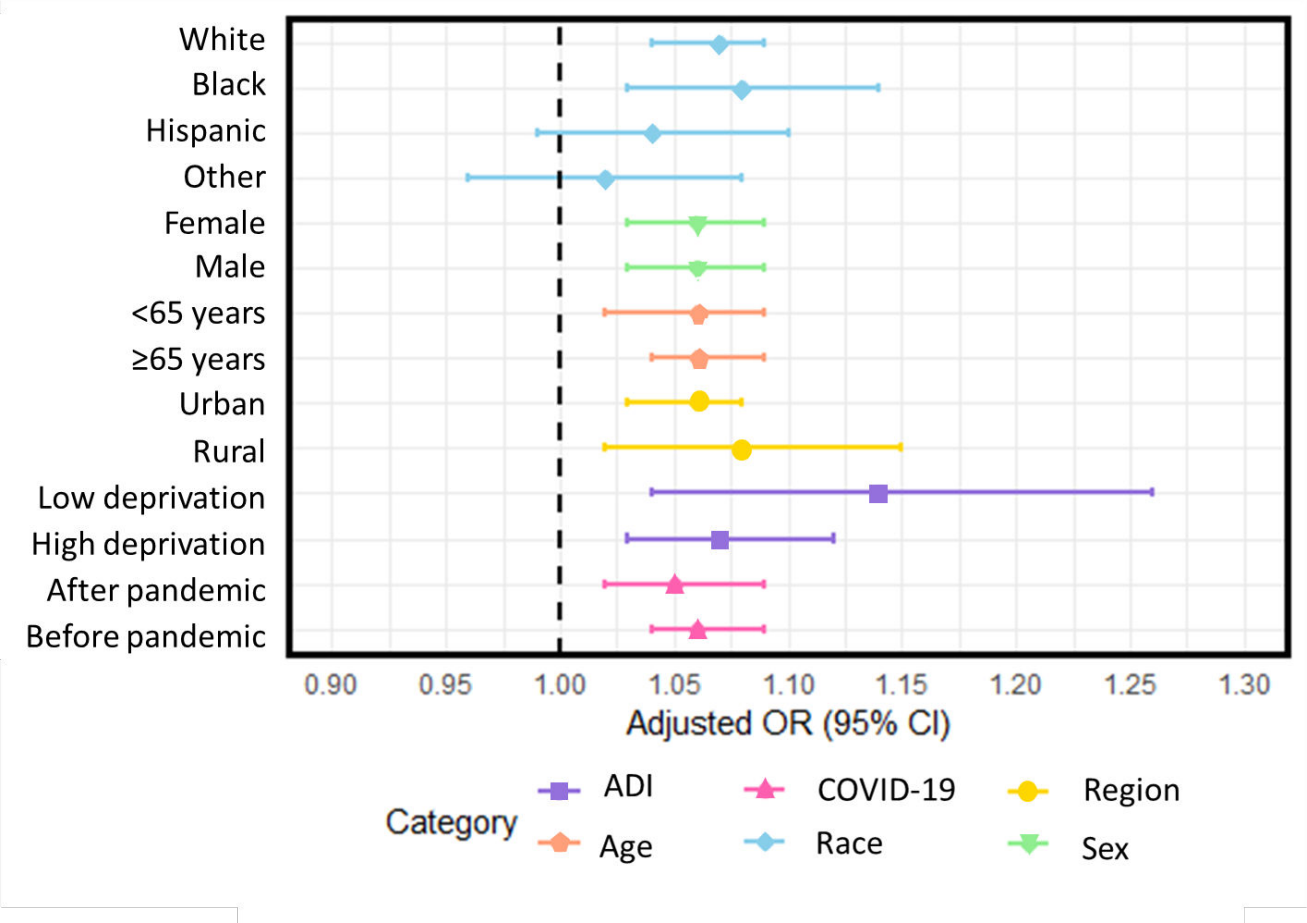
Results for multivariate logistic regressions and subgroup analysis for monthly (May) maximum temperature (Fahrenheit). ADI: Area Deprivation Index; OR: odds ratio.

Subgroup analyses were conducted to examine the association between May maximum temperature and the composite outcome of 1-year HF readmission and mortality, stratified by race, sex, age group, COVID-19 pandemic period, rurality, and ADI. In our subgroup analysis of the association between May maximum temperature and the outcome, we observed consistent trends across most subgroups, although not all were statistically significant. Among racial groups, the aORs were statistically significant for White (aOR 1.07, 95% CI 1.04-1.09) and Black patients (aOR 1.08, 95% CI 1.03-1.14), indicating a meaningful association in these populations. However, the association was not statistically significant for Hispanic (aOR 1.04, 95% CI 0.99-1.10) or other race groups (aOR 1.02, 95% CI 0.96-1.08). Both female (aOR 1.06, 95% CI 1.03-1.09) and male (aOR 1.06, 95% CI 1.03-1.09) subgroups showed statistically significant associations with the outcome, as did both age groups: individuals aged <65 years (aOR 1.06, 95% CI 1.02-1.09) and those aged ≥65 years (aOR 1.06, 95% CI 1.04-1.09) showed similar effect sizes. Regional differences were also significant, with both urban (aOR 1.06, 95% CI 1.03-1.08) and rural regions (aOR 1.08, 95% CI 1.02-1.15) displaying associations with the outcome. For ADI, individuals in areas of low deprivation showed a significant association (aOR 1.14, 95% CI 1.04-1.26), and those in areas of high deprivation also exhibited a statistically significant association (aOR 1.07, 95% CI 1.03-1.12). In addition, the COVID-19 period analysis indicated a significant association both before the pandemic (aOR 1.06, 95% CI 1.04-1.09) and after the pandemic began (aOR 1.05, 95% CI 1.02-1.09). This detailed breakdown suggests that a higher maximum temperature in May was consistently linked to an increased risk of the adverse composite outcomes for patients with HF, independent of their race, age, or socioeconomic status (SES) (Table 4; Figure 2).

**Table 4.** Results from multivariate logistic regression and subgroup analysis for monthly (May) maximum temperature (Fahrenheit).

Variable and subgroup		Adjusted OR^a^ (95% CI)
Overall		1.04 (1.02-1.06)
Race		
White		1.07 (1.04-1.09)
Black		1.08 (1.03-1.14)
Hispanic		1.04 (0.99-1.10)
Other		1.02 (0.96-1.08)
Sex		
Female		1.06 (1.03-1.09)
Male		1.06 (1.03-1.09)
Age (years)		
<65		1.06 (1.02-1.09)
≥65		1.06 (1.04-1.09)
Region		
Urban		1.06 (1.03-1.08)
Rural		1.08 (1.02-1.15)
ADI^b^		
Low deprivation		1.14 (1.04-1.26)
High deprivation		1.07 (1.03-1.12)
COVID-19		
After pandemic		1.05 (1.02-1.09)
Before pandemic		1.06 (1.04-1.09)

a OR: odds ratio.

b ADI: Area Deprivation Index.

Furthermore, we tested the association between May maximum temperature and the 1-year readmission outcome and observed similar trends, with an aOR of 1.05 (95% CI 1.03-1.07). These results suggest a consistent association between elevated May temperatures and adverse outcomes in patients with HF, reinforcing the robustness of elevated temperature as a positive risk factor for higher HF-related adverse outcomes.

## Discussion

### Principal Findings

Using the standard 2-phase EWAS-MLR approach, we assessed the association of 1308 external exposome variables in relation to 1-year HF readmission and mortality, leveraging real-world data. We discovered that an elevated maximum May temperature was significantly associated with an increased risk of HF readmission and mortality in Florida.

Concerns have been raised about the interaction between extreme temperatures and factors such as SES, urban-rural differences, and greenspace availability. Populations in higher-SES areas may experience less impact from high temperatures due to better access to air conditioning and health care, mitigating some of the heat’s effects [57]. To explore this, we conducted subgroup analyses by SES (using the ADI) and by urban versus rural areas. Our results showed that none of the subgroup variables, including sex, rurality, SES, and COVID-19 period, significantly modified the association between temperature and HF outcomes. This consistency suggests that temperature exerts a direct, uniform impact on HF one-year readmission and mortality, regardless of socioeconomic differences. Additional subgroup analysis revealed that higher maximum temperatures in May were consistently associated with an increased risk of HF 1-year readmission and mortality across most groups, with significant associations observed among White and Black patients, both sexes, and age groups. This consistency suggests that elevated May temperatures pose a broad risk to patients with HF, largely independent of demographic or socioeconomic factors.

### Comparison With Prior Work

To our knowledge, this retrospective cohort study is the first to evaluate the impact of a comprehensive array of contextual-level external exposome factors, derived from patients’ residential histories, on HF 1-year readmission and mortality among patients with HHF. Our findings align with previous studies on the impact of temperature and climate on cardiovascular disease (CVD) outcomes, which have consistently highlighted a complex relationship, with both extreme heat and cold temperatures being associated with increased risks of various CVD adverse outcomes [58,59]. For example, a study by Lopez et al [58] found that mortality in chronic HF exhibits a seasonal pattern, with a 13.9% higher all-cause mortality rate in winter than in summer. On the other hand, Chan et al [60] found that an average 1°C increase in daily mean temperature above 28.2°C was associated with a 1.8% increase in mortality, with higher vulnerability observed among women, men younger than 75 years, individuals in low-SES districts, those with unknown residence, married people, and deaths from cardiovascular and respiratory infections. However, differences emerge in the magnitude of risk associated with specific temperature thresholds, which can vary by geographic location, population acclimatization, and study methodologies [59,61-63]. Our findings align with previous studies that an increasing level of heat significantly elevates the risk of CVD-related mortality and morbidity, with pronounced risks for residents of tropical climates [62]. Considering Florida’s subtropical environment, which experiences relatively mild winters, the impact of low temperatures on HF outcomes in this setting may be limited.

The potential biological plausibility behind the impact of heat on adverse outcomes of patients with HF can be attributed to several mechanisms. Heat exposure can lead to dehydration and increased blood viscosity, placing additional strain on the cardiovascular system [64]. It can also induce systemic inflammation and oxidative stress, which are known contributors to atherosclerosis and other CVDs [64,65]. Furthermore, heat can exacerbate the body’s stress response, increasing heart rate and blood pressure, thereby imposing further risk on individuals with pre-existing cardiovascular conditions [66]. However, previous research by Nam et al [67] has highlighted that increased potassium use during summer months showed a protective effect against all-cause mortality increases with higher temperatures. Understanding these mechanisms is crucial for developing strategies to mitigate adverse outcomes in patients with HHF residing in high-temperature environments.

### Limitations

This study has limitations. First, the generalizability of our findings may be limited due to the regional focus of the study. The majority of our cohort population resided in Florida, and as such, our observations may not readily extend to other states with different climatic conditions. Our study did not reveal a notable impact of cold temperatures, as prior studies have, which is likely attributable to Florida’s lack of low-temperature winters [63,68]. Future studies using nationally representative data sources should investigate the consistency of these associations across more diverse environmental settings. Second, the EHR data are limited to individuals who made health care encounters within the participating networks, so there would be a chance that we failed to capture HF readmission or mortality that occurred outside of the participating health care systems, which would lead to misclassification in the nonoutcome group. To minimize loss to follow-up for longer-term outcomes, we limited the study follow-up period to one year. Considering that the observed association remains significant despite potential misclassification in the nonoutcome group, this suggests that the effect of maximum temperature in May on adverse HF outcomes may be even stronger than reported, underscoring its potential impact on HR-related adverse outcome risk. Third, we acknowledge that using county-level exposures may overlook intracounty variations that could provide more localized environmental and social insights into HR outcomes. Unfortunately, our data were not available at a finer spatial granularity, such as ZIP code or census tract, which may have limited our ability to detect these localized effects. Future studies with access to more detailed geographic data could offer deeper insights into the impact of environmental exposures on HR, allowing for targeted interventions at the community level. Fourth, our selection of external exposome factors may not fully encompass the wide range of social determinants of health that impact adverse HF outcomes. Future studies could enhance understanding by incorporating additional environmental variables, such as walkability and data from the EPA’s Smart Location Database, to provide a more comprehensive view of how the built environment influences health outcomes. Furthermore, our analysis was limited to exposure data available only through 2019, which prevented us from including pandemic-era data (2020‐2022) for variables such as employment, economic resilience, and air quality. Due to a typical 2‐5-year delay in accessing contextual social risk data, we believe these contextual social factors likely remained relatively stable over this short period, minimizing potential effects on our findings. In addition, our effect of the exposome is not quantified as aggregated exposure. While an aggregate exposure approach—combining individual- and contextual-level measures—would ideally capture a more comprehensive picture of the factors affecting cardiovascular outcomes, implementing such a method typically requires a preselected study cohort and prospective data collection. This process can be costly, time-intensive, and may restrict both the sample size and the generalizability of findings. Moreover, measuring temperature by the maximum temperature in May limits our ability to capture the day-to-day and month-to-month temperature fluctuations, which could affect HF outcomes. As a result, our study may not fully reflect the nuanced relationship between short-term temperature variation and adverse health outcomes. Furthermore, as an observational study, our analysis is inherently limited in its ability to rule out unmeasured confounding factors, which may influence the observed associations. Although we have applied rigorous statistical methods and adjusted for known confounders, the possibility of residual confounding remains. Future research, including randomized controlled trials or studies with more comprehensive confounder adjustment, could provide stronger causal evidence.

### Conclusions

In summary, in a cohort of HHF patients from OneFlorida+ Network, we identified that elevated maximum temperatures in May were consistently associated with an increased risk of 1-year HF readmission and mortality. This association remained significant across various subgroups, including race, sex, age, SES, and urban-rural residence, suggesting that high temperatures in late spring pose a uniform risk to patients with HF, regardless of demographic or socioeconomic background. Our study has important implications for the impact of global warming and environmental factors on HF outcomes. Given the projected increases in global temperatures, further investigations are warranted to uncover the intricate mechanisms through which extreme heat potentially influences adverse HF outcomes and to develop targeted strategies to mitigate these risks in affected populations. Clinicians should be aware of temperature-related risk, particularly in regions with pronounced seasonal temperature variations, and consider environmental conditions when planning HF management and follow-up care.

## Supplementary material

10.2196/71595 Multimedia Appendix 1 Supplementary tables and figures.

10.2196/71595 Checklist 1 STROBE (Strengthening the Reporting of Observational Studies in Epidemiology) checklist.

## Abbreviations

ADI: Area Deprivation Index
aOR: adjusted odds ratio
COPD: chronic obstructive pulmonary disease
CRT: cardiac resynchronization therapy
CVD: cardiovascular disease
EHR: electronic health record
ExWAS: external exposome-wide association study
HF: heart failure
HFpEF: heart failure with preserved ejection fraction
HHF: hospitalized for heart failure
ICD: implantable cardioverter-defibrillator
*ICD-10*: *International Classification of Diseases, Tenth Revision*
*ICD-10-CM*: *International Classification of Diseases: Tenth Revision: Clinical Modification*
*ICD-9-CM*: *International Classification of Diseases: Ninth Revision: Clinical Modification*
LASSO: least absolute shrinkage and selection operator
SES: socioeconomic status
SNAP: Supplemental Nutrition Assistance Program
STROBE: Strengthening the Reporting of Observational Studies in Epidemiology
