# Supplementary material for: External Exposome Factors and Adverse Heart Failure Outcomes in the OneFlorida+ Network: Retrospective Cohort Study
Source: JMIR Form Res. 2025 Aug 25;9:e71595. doi: 10.2196/71595 (PMC12377874; doi:10.2196/71595)
Supplement: Multimedia Appendix 1 [file formative-v9-e71595-s001.docx]

**Figure S1.** ExWAS flowchart.

**
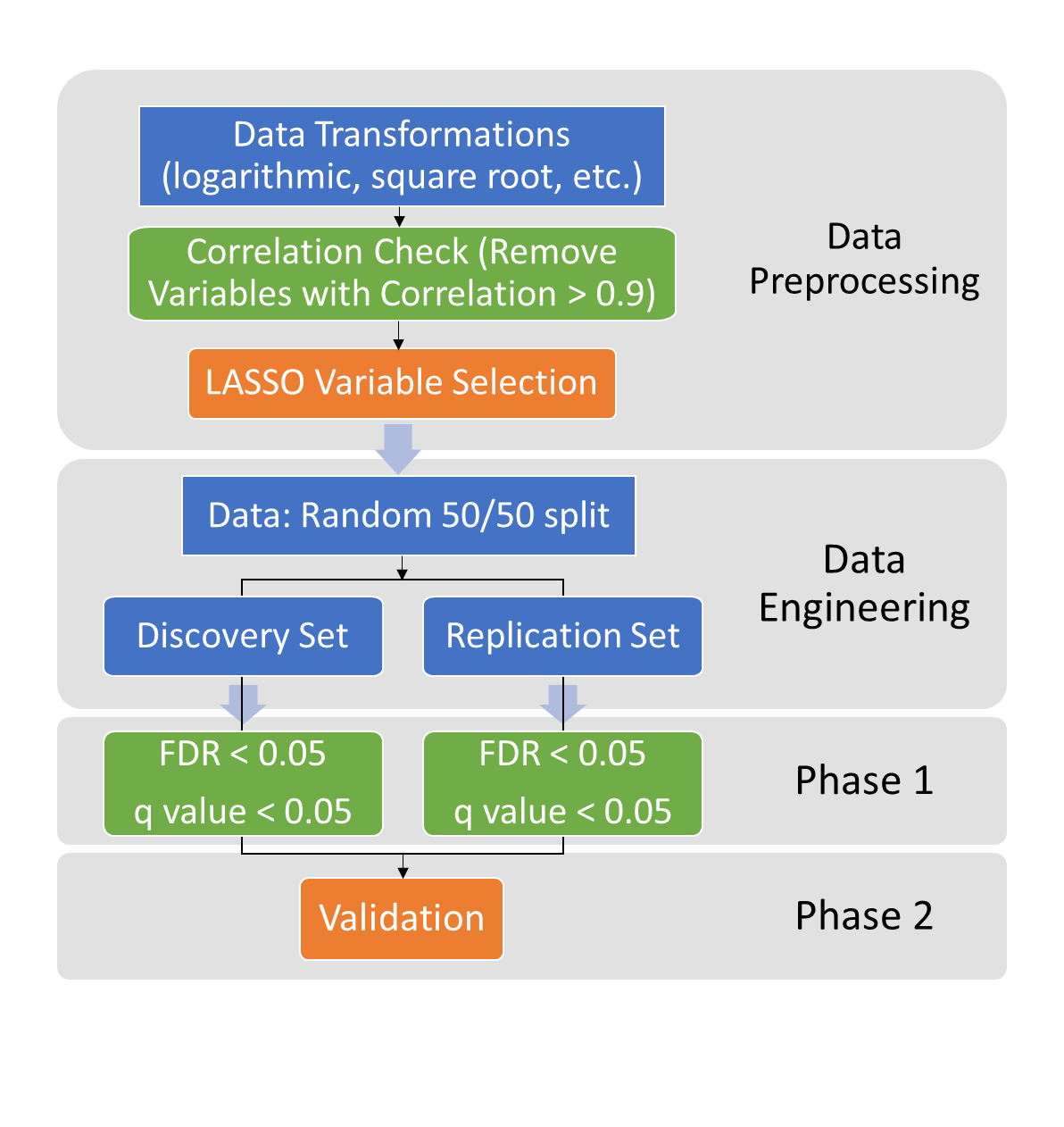
**

**Figure S2.** Patient flowchart.

**
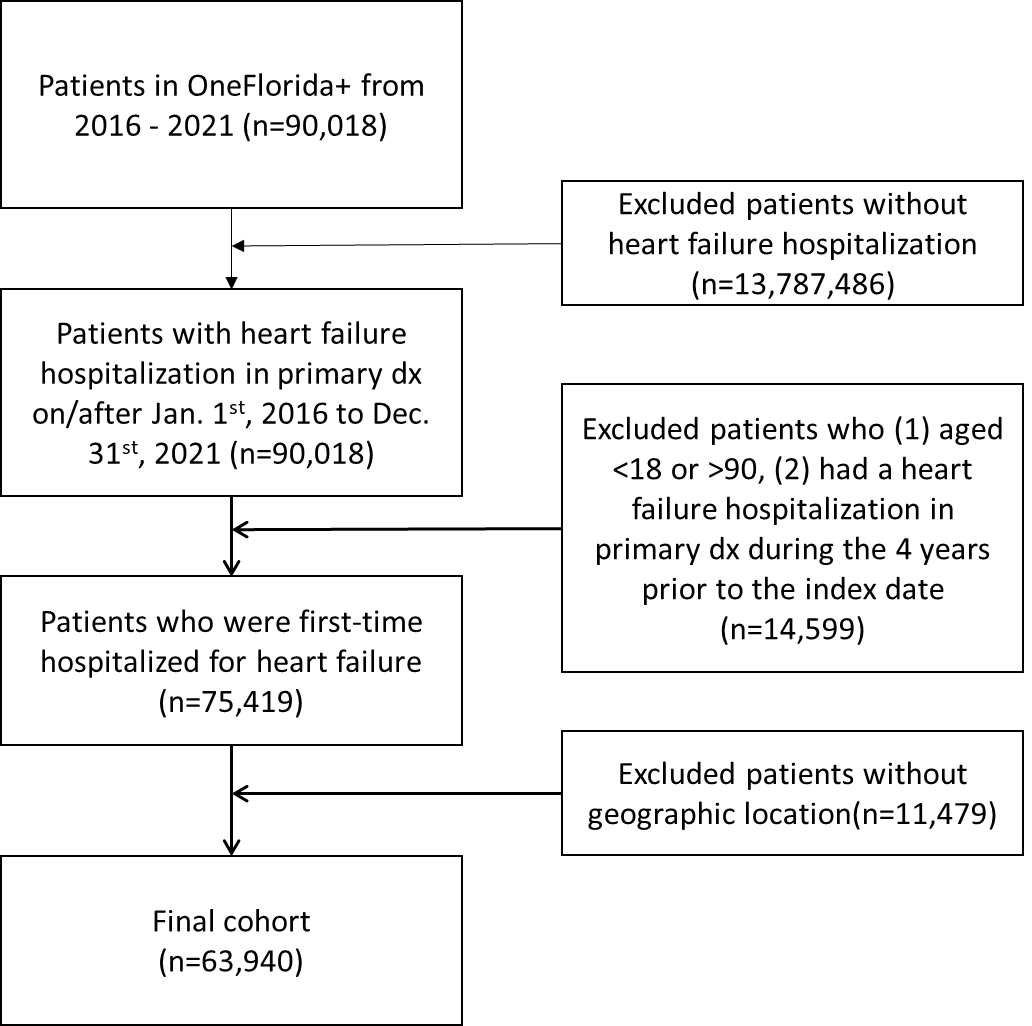
**

**Figure S3.** Volcano plot showing the results from Phase 1 of the external ExWAS of HHF and death.


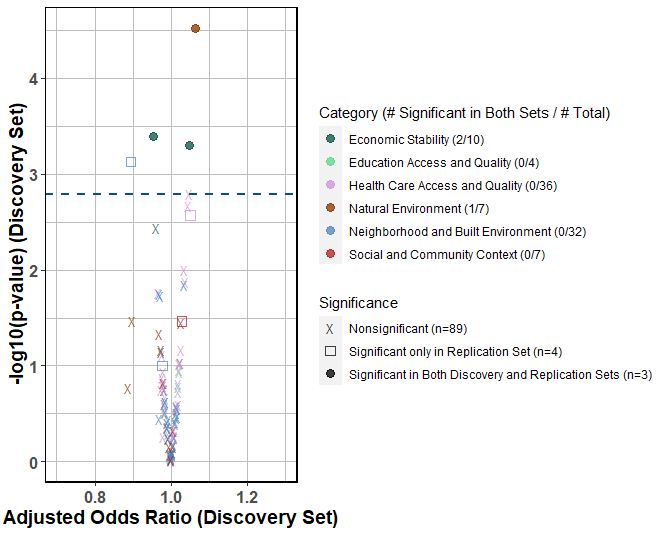


**Figure S4.** Correlation between phase 1 variables.


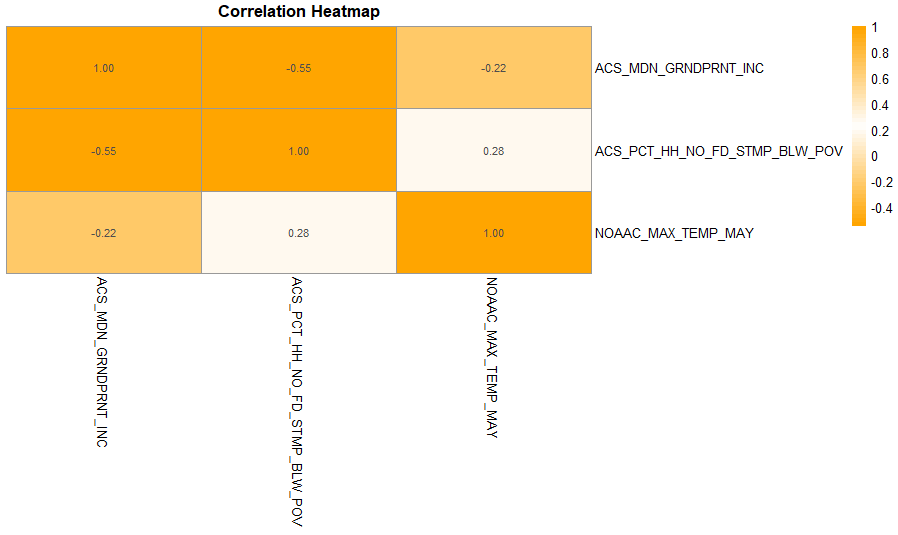


**Table S1.** Comorbidities identification.

| Comorbidities | ICD | Procedure codes |
| --- | --- | --- |
| Heart failure with preserved ejection fraction | ICD-9: 428.3;  ICD-10: I50.3; |  |
| Chronic obstructive pulmonary disease | ICD-9: 490, 491.0, 491.1, 491.8, 491.9, 492.0, 492.8, 491.20, 491.21, 491.22, 494.0, 494.1, 496;  ICD-10: J40, J41.0, J41.1, J41.8, J42, J43.0, J43.1, J43.2, J43.8, J43.9, J44.0, J44.1, J44.9, J47.0, J47.1, J47.9; |  |
| Myocardial infarction | ICD-9: 412;  ICD-10: I252; |  |
| Anemia | ICD-9: 280.0, 280.1, 280.8, 280.9, 281.0, 281.1, 281.2, 281.3, 281.4, 281.8, 281.9, 282.0, 282.1, 282.2, 282.3, 282.40, 282.41, 282.42, 282.43, 282.44, 282.45, 282.46, 282.47, 282.49, 282.5, 282.60, 282.61, 282.62, 282.63, 282.64, 282.68, 282.69, 282.7, 282.8, 282.9, 283.0, 283.10, 283.11, 283.19, 283.2, 283.9, 284.01, 284.09, 284.11, 284.12, 284.19, 284.2, 284.81, 284.89, 284.9, 285.0, 285.1, 285.21, 285.22, 285.29, 285.3, 285.8, 285.9;  ICD-10: D50.0, D50.1, D50.8, D50.9, D51.0, D51.1, D51.2, D51.3, D51.8, D51.9, D52.0, D52.1, D52.8, D52.9, D53.0, D53.1, D53.2, D53.8, D53.9, D55.0, D55.1, D55.2, D55.3, D55.8, D55.9, D56.0, D56.1, D56.2, D56.3, D56.4, D56.5, D56.8, D56.9, D57.00, D57.01, D57.02, D57.1, D57.20, D57.211, D57.212, D57.219, D57.3, D57.40, D57.411, D57.412, D57.419, D57.80, D57.811, D57.812, D57.819, D58.0, D58.1, D58.2, D58.8, D58.9, D59.0, D59.1, D59.2, D59.3, D59.4, D59.5, D59.6, D59.8, D59.9, D60.0, D60.1, D60.8, D60.9, D61.01, D61.09, D61.1, D61.2, D61.3, D61.810, D61.811, D61.818, D61.82, D61.89, D61.9, D62, D63.0, D63.1, D63.8, D64.0, D64.1, D64.2, D64.3, D64.4, D64.81, D64.89, D64.9; |  |
| Implantable cardioverter-defibrillator and/or cardiac resynchronization therapy | ICD-9: V45.0;  ICD-10: Z95.0; | CPT: 33224, 33225, 33226, 33206, 33207, 33208, 33214, 33227, 33228, 33229, 33212, 33213, 33221;  ICD-10-CM: Z95.810, Z95.0;  ICD-9-CM: V45.02, V45.01 |
| Diabetes | ICD-9: 249.00, 249.01, 249.10, 249.11, 249.20, 249.21, 249.30, 249.31, 249.40, 249.41, 249.50, 249.51, 249.60, 249.61, 249.70, 249.71, 249.80, 249.81, 249.90, 249.91, 250.00, 250.01, 250.02, 250.03, 250.10, 250.11, 250.12, 250.13, 250.20, 250.21, 250.22, 250.23, 250.30, 250.31, 250.32, 250.33, 250.40, 250.41, 250.42, 250.43, 250.50, 250.51, 250.52, 250.53, 250.60, 250.61, 250.62, 250.63, 250.70, 250.71, 250.72, 250.73, 250.80, 250.81, 250.82, 250.83, 250.90, 250.91, 250.92, 250.93, 357.2, 362.01, 362.02, 362.03, 362.04, 362.05, 362.06, 366.41;  ICD-10: E08.00, E08.01, E08.10, E08.11, E08.21, E08.22, E08.29, E08.311, E08.319, E08.321, E08.3211, E08.3212, E08.3213, E08.3219, E08.329, E08.3291, E08.3292, E08.3293, E08.3299, E08.331, E08.3311, E08.3312, E08.3313, E08.3319, E08.339, E08.3391, E08.3392, E08.3393, E08.3399, E08.341, E08.3411, E08.3412, E08.3413, E08.3419, E08.349, E08.3491, E08.3492, E08.3493, E08.3499, E08.351, E08.3511, E08.3512, E08.3513, E08.3519, E08.3521, E08.3522, E08.3523, E08.3529, E08.3531, E08.3532, E08.3533, E08.3539, E08.3541, E08.3542, E08.3543, E08.3549, E08.3551, E08.3552, E08.3553, E08.3559, E08.359, E08.3591, E08.3592, E08.3593, E08.3599, E08.36, E08.37X1, E08.37X2, E08.37X3, E08.37X9, E08.39, E08.40, E08.41, E08.42, E08.43, E08.44, E08.49, E08.51, E08.52, E08.59, E08.610, E08.618, E08.620, E08.621, E08.622, E08.628, E08.630, E08.638, E08.641, E08.649, E08.65, E08.69, E08.8, E08.9, E09.00, E09.01, E09.10, E09.11, E09.21, E09.22, E09.29, E09.311, E09.319, E09.321, E09.3211, E09.3212, E09.3213, E09.3219, E09.329, E09.3291, E09.3292, E09.3293, E09.3299, E09.331, E09.3311, E09.3312, E09.3313, E09.3319, E09.339, E09.3391, E09.3392, E09.3393, E09.3399, E09.341, E09.3411, E09.3412, E09.3413, E09.3419, E09.349, E09.3491, E09.3492, E09.3493, E09.3499, E09.351, E09.3511, E09.3512, E09.3513, E09.3519, E09.3521, E09.3522, E09.3523, E09.3529, E09.3531, E09.3532, E09.3533, E09.3539, E09.3541, E09.3542, E09.3543, E09.3549, E09.3551, E09.3552, E09.3553, E09.3559, E09.359, E09.3591, E09.3592, E09.3593, E09.3599, E09.36, E09.37X1, E09.37X2, E09.37X3, E09.37X9, E09.39, E09.40, E09.41, E09.42, E09.43, E09.44, E09.49, E09.51, E09.52, E09.59, E09.610, E09.618, E09.620, E09.621, E09.622, E09.628, E09.630, E09.638, E09.641, E09.649, E09.65, E09.69, E09.8, E09.9, E10.10, E10.11, E10.21, E10.22, E10.29, E10.311, E10.319, E10.321, E10.3211, E10.3212, E10.3213, E10.3219, E10.329, E10.3291, E10.3292, E10.3293, E10.3299, E10.331, E10.3311, E10.3312, E10.3313, E10.3319, E10.339, E10.3391, E10.3392, E10.3393, E10.3399, E10.341, E10.3411, E10.3412, E10.3413, E10.3419, E10.349, E10.3491, E10.3492, E10.3493, E10.3499, E10.351, E10.3511, E10.3512, E10.3513, E10.3519, E10.3521, E10.3522, E10.3523, E10.3529, E10.3531, E10.3532, E10.3533, E10.3539, E10.3541, E10.3542, E10.3543, E10.3549, E10.3551, E10.3552, E10.3553, E10.3559, E10.359, E10.3591, E10.3592, E10.3593, E10.3599, E10.36, E10.37X1, E10.37X2, E10.37X3, E10.37X9, E10.39, E10.40, E10.41, E10.42, E10.43, E10.44, E10.49, E10.51, E10.52, E10.59, E10.610, E10.618, E10.620, E10.621, E10.622, E10.628, E10.630, E10.638, E10.641, E10.649, E10.65, E10.69, E10.8, E10.9, E11.00, E11.01, E11.10, E11.11, E11.21, E11.22, E11.29, E11.311, E11.319, E11.321, E11.3211, E11.3212, E11.3213, E11.3219, E11.329, E11.3291, E11.3292, E11.3293, E11.3299, E11.331, E11.3311, E11.3312, E11.3313, E11.3319, E11.339, E11.3391, E11.3392, E11.3393, E11.3399, E11.341, E11.3411, E11.3412, E11.3413, E11.3419, E11.349, E11.3491, E11.3492, E11.3493, E11.3499, E11.351, E11.3511, E11.3512, E11.3513, E11.3519, E11.3521, E11.3522, E11.3523, E11.3529, E11.3531, E11.3532, E11.3533, E11.3539, E11.3541, E11.3542, E11.3543, E11.3549, E11.3551, E11.3552, E11.3553, E11.3559, E11.359, E11.3591, E11.3592, E11.3593, E11.3599, E11.36, E11.37X1, E11.37X2, E11.37X3, E11.37X9, E11.39, E11.40, E11.41, E11.42, E11.43, E11.44, E11.49, E11.51, E11.52, E11.59, E11.610, E11.618, E11.620, E11.621, E11.622, E11.628, E11.630, E11.638, E11.641, E11.649, E11.65, E11.69, E11.8, E11.9, E13.00, E13.01, E13.10, E13.11, E13.21, E13.22, E13.29, E13.311, E13.319, E13.321, E13.3211, E13.3212, E13.3213, E13.3219, E13.329, E13.3291, E13.3292, E13.3293, E13.3299, E13.331, E13.3311, E13.3312, E13.3313, E13.3319, E13.339, E13.3391, E13.3392, E13.3393, E13.3399, E13.341, E13.3411, E13.3412, E13.3413, E13.3419, E13.349, E13.3491, E13.3492, E13.3493, E13.3499, E13.351, E13.3511, E13.3512, E13.3513, E13.3519, E13.3521, E13.3522, E13.3523, E13.3529, E13.3531, E13.3532, E13.3533, E13.3539, E13.3541, E13.3542, E13.3543, E13.3549, E13.3551, E13.3552, E13.3553, E13.3559, E13.359, E13.3591, E13.3592, E13.3593, E13.3599, E13.36, E13.39, E13.40, E13.41, E13.42, E13.43, E13.44, E13.49, E13.51, E13.52, E13.59, E13.610, E13.618, E13.620, E13.621, E13.622, E13.628, E13.630, E13.638, E13.641, E13.649, E13.65, E13.69, E13.8, E13.9; |  |
| Breast cancer | ICD-9: 174.0, 174.1, 174.2, 174.3, 174.4, 174.5, 174.6, 174.8, 174.9, 175.0, 175.9, 233.0, V10.3;  ICD-10: C50.011, C50.012, C50.019, C50.021, C50.022, C50.029, C50.111, C50.112, C50.119, C50.121, C50.122, C50.129, C50.211, C50.212, C50.219, C50.221, C50.222, C50.229, C50.311, C50.312, C50.319, C50.321, C50.322, C50.329, C50.411, C50.412, C50.419, C50.421, C50.422, C50.429, C50.511, C50.512, C50.519, C50.521, C50.522, C50.529, C50.611, C50.612, C50.619, C50.621, C50.622, C50.629, C50.811, C50.812, C50.819, C50.821, C50.822, C50.829, C50.911, C50.912, C50.919, C50.921, C50.922, C50.929, D05.00, D05.01, D05.02, D05.10, D05.11, D05.12, D05.80, D05.81, D05.82, D05.90, D05.91, D05.92, Z85.3; |  |
| Colorectal cancer | ICD-9: 153.0, 153.1, 153.2, 153.3, 153.4, 153.5, 153.6, 153.7, 153.8, 153.9,154.0,154.1, 230.3, 230.4, V10.05, V10.06;  ICD-10: C18.0, C18.1, C18.2, C18.3, C18.4, C18.5, C18.6, C18.7, C18.8, C18.9, C19, C20, D01.0, D01.1, D01.2, Z85.038, Z85.040, Z85.048; |  |
| Prostate cancer | ICD-9: 185, 233.4, V10.46;  ICD-10: C61, D07.5, Z85.46; |  |
| Lung cancer | ICD-9: 162.2, 162.3, 162.4, 162.5, 162.8, 162.9, 231.2, V10.11;  ICD-10: C34.00, C34.01, C34.02, C34.10, C34.11, C34.12, C34.2, C34.30, C34.31, C34.32, C34.80, C34.81, C34.82, C34.90, C34.91, C34.92, D02.20, D02.21, D02.22, Z85.110, Z85.118; |  |
| Endometrial cancer | ICD-9: 182.0, 233.2, V10.42;  ICD-10: C54.1, C54.2, C54.3, C54.8, C54.9, D07.0, Z85.42; |  |

**Table S2.** The chosen transformations and parameters for all the LASSO-selected external exposome variables.

| Variable name | Transformation | Category | Notes |
| --- | --- | --- | --- |
| ACS_MDN_GRNDPRNT_INC | sqrt_x | Economic Stability | Median income of grandparent householder and/or spouse responsible for grandchildren under 18 (dollars, inflation-adjusted to data file year) |
| ACS_MEDIAN_HH_INC_AIAN | no_transform | Economic Stability | Median household income for households with an American Indian and Alaska Native alone householder (dollars, inflation-adjusted to data file year) |
| ACS_MEDIAN_HH_INC_NHPI | no_transform | Economic Stability | Median household income for households with a Native Hawaiian and other Pacific Islander alone householder (dollars, inflation-adjusted to data file year) |
| ACS_MEDIAN_YEAR_BUILT | no_transform | Neighborhood and Built Environment | Median year structure built of housing units |
| ACS_PCT_AIAN | center_scale | Social and Community Context | Percentage of population reporting American Indian and Alaska Native race alone |
| ACS_PCT_AIAN_COMB | log_x | Social and Community Context | Percentage of population reporting American Indian and Alaska Native alone or in combination with one or more races |
| ACS_PCT_AIAN_MALE | yeojohnson | Social and Community Context | Percentage of American Indian and Alaska Native alone population reporting male |
| ACS_PCT_DRIVE_2WORK | center_scale | Neighborhood and Built Environment | Percentage of workers taking a car, truck, or van to work (ages 16 and over) |
| ACS_PCT_HH_NO_FD_STMP_BLW_POV | yeojohnson | Economic Stability | Percentage of households not receiving food stamps/SNAP with income below the poverty level |
| ACS_PCT_HU_OTHER | exp_x | Neighborhood and Built Environment | Percentage of occupied housing units with other heating fuel |
| ACS_PCT_MANUFACT | sqrt_x | Economic Stability | Percentage of employed working in manufacturing (ages 16 and over) |
| ACS_PCT_MULT_RACE_MALE | center_scale | Social and Community Context | Percentage of population reporting two or more races and male |
| ACS_PCT_NO_WORK_NO_SCHL_16_19 | yeojohnson | Education Access and Quality | Percentage of teens and adults who are unemployed and not in school (between ages 16 and 19) |
| ACS_PCT_POP_SAME_SEX_UNMRD_P | no_transform | Social and Community Context | Percentage of population with same-sex unmarried partner |
| ACS_PCT_POV_AIAN | arcsinh_x | Economic Stability | Percentage of American Indian or Alaska Native population below poverty level |
| ACS_PCT_POV_BLACK | arcsinh_x | Economic Stability | Percentage of Black or African American population below poverty level |
| ACS_PCT_TAXICAB_2WORK | yeojohnson | Neighborhood and Built Environment | Percentage of workers taking taxicab, motorcycle, bicycle, or other means to work (ages 16 and over) |
| ACS_PCT_TRANSPORT | center_scale | Economic Stability | Percentage of employed working in transportation and warehousing, and in utilities (ages 16 and over) |
| ACS_PCT_VET_UNEMPL_18_64 | arcsinh_x | Economic Stability | Percentage of civilian veterans that are unemployed (between ages 18 and 64) |
| ACS_PCT_WHITE_FEMALE | center_scale | Social and Community Context | Percentage of White non-Hispanic alone population reporting female |
| AHA_HHI_SHRTTRM_ACUTE_ADMSN_CTY | center_scale | Health Care Access and Quality | Herfindahl-Hirschman Index for short-term acute care hospitals (admission, County level) |
| AHRF_ARBRNE_ST_G_ISO_HOSPS_RATE | log_x | Health Care Access and Quality | Total number of short-term general hospitals with airborne infection isolation rooms per 1,000 population |
| AHRF_GASTROENTEROLOGY_RATE | log_x | Health Care Access and Quality | Total number of gastroenterology specialists per 1,000 population |
| AHRF_GEN_INTERNAL_MED_RATE | exp_x | Health Care Access and Quality | Total number of non-federal general internal medicine physicians per 1,000 population |
| AHRF_HOSP_TEACHING | log_x | Health Care Access and Quality | Total number of teaching hospitals |
| AHRF_HOSP_TELE_STROKE_RATE | log_x | Health Care Access and Quality | Total number of hospitals with telehealth stroke care per 1,000 population |
| AHRF_HOSPS_RATE | log_x | Health Care Access and Quality | Total number of hospitals per 1,000 population |
| AHRF_NURSE_MIDWIVES_RATE | log_x | Health Care Access and Quality | Total number of advanced practice nurse midwives with NPI per 1,000 population |
| AHRF_PHYSICIAN_ASSIST_RATE | exp_x | Health Care Access and Quality | Total number of physician assistants with NPI per 1,000 population |
| AHRF_RADI_RATE | log_x | Health Care Access and Quality | Total number of non-federal radiologists per 1,000 population |
| AHRF_TOT_NH_BED_STNGH | center_scale | Health Care Access and Quality | Total number of short-term non-general hospital nursing home beds |
| AMFAR_HIVTFAC_RATE | log_x | Health Care Access and Quality | Total number of substance abuse facilities offering HIV testing per 1,000 population |
| AMFAR_MEDHIVTFAC_RATE | sqrt_x | Health Care Access and Quality | Total number of substance abuse facilities offering HIV testing and accept Medicaid per 1,000 population |
| AMFAR_MHFAC_RATE | log_x | Health Care Access and Quality | Total number of facilities that provide mental health services per 1,000 population |
| AMFAR_TOT_MHFAC | log_x | Health Care Access and Quality | Total number of facilities that provide mental health services |
| AQI_MEDIAN | no_transform | Neighborhood and Built Environment | Median AQI |
| AQI_UNHEALTHY | exp_x | Neighborhood and Built Environment | Unhealthy Days |
| AQS_SO2_99_PCT | yeojohnson | Neighborhood and Built Environment | For Sulfur Dioxide, the 99th percentile of the daily max 1-hour measurements in the year. |
| CCBP_BWLSTORES_RATE | exp_x | Neighborhood and Built Environment | Total number of beer, wine and liquor stores per 1,000 people |
| CCBP_GAMBLING_RATE | no_transform | Neighborhood and Built Environment | Total number of gambling establishments per 1,000 people |
| CCBP_LAB_RATE | log_x | Neighborhood and Built Environment | Total number of medical and diagnostic laboratories per 1,000 people |
| CCBP_RET_RATE | log_x | Neighborhood and Built Environment | Total number of continuing care retirement communities and assisted living facilities for the elderly per 1,000 people |
| CCD_FED_REVENUE_CNA | log_x | Education Access and Quality | Federal revenue distributed by state through Child Nutrition Act (Dollars) per student |
| CCD_STATE_REVENUE_LUNCH | yeojohnson | Education Access and Quality | State revenue from school lunch programs (Dollars) per student |
| CCD_TOT_REVENUE | boxcox | Education Access and Quality | Total revenue (Dollars) per student |
| CDCAP_GONORRHEA_RATE | yeojohnson | Health Care Access and Quality | Total number of confirmed gonorrhea diagnoses per 100,000 population |
| CDCAP_HIVDIAG_F_RATE_ABOVE13 | log_x | Health Care Access and Quality | Total number of females living with diagnosed HIV at the end of the year per 100,000 (ages 13 and over) |
| CDCAP_TUBERCULOSIS_RATE | center_scale | Health Care Access and Quality | Total number of tuberculosis diagnoses per 100,000 population |
| CDCP_BLOOD_MED_ADULT_A | yeojohnson | Health Care Access and Quality | Age-adjusted prevalence of adults aged 18 years and older with high blood pressure taking medicine for high blood pressure control (%) |
| CHRD_CHILD_POV_ASIAN | boxcox | Economic Stability | Percentage of Asian/Pacific Islander children (under age 18) living in poverty - from the 2014-2018 ACS |
| CHRD_DRIVE_ALONE | sqrt_x | Neighborhood and Built Environment | Percentage of workers who drive alone to work |
| CHRD_DRIVE_ALONE_ASIAN | yeojohnson | Neighborhood and Built Environment | Percentage of Asian/Pacific Islander workers who drive alone to work |
| CHRD_LBW_ASIAN | sqrt_x | Health Care Access and Quality | Percentage of births with low birthweight (<2500g) for non-Hispanic Asians |
| CHRD_PCT_WITHACCESS | log_x | Neighborhood and Built Environment | Percentage of the population with access to places for physical activity |
| CHRD_PHR_HISPAN | no_transform | Health Care Access and Quality | Discharges for Ambulatory Care Sensitive Conditions per 100,000 Medicare Enrollees for Hispanics |
| CHRD_VAX_WHITE | no_transform | Health Care Access and Quality | Percentage of annual Medicare enrollees having an annual flu vaccination for Whites |
| EPEST_HIGH_KG_CYPRODINIL | log_x | Neighborhood and Built Environment | Estimated Annual Agricultural Pesticide Use - CYPRODINIL |
| EPEST_HIGH_KG_DICAMBA | arcsinh_x | Neighborhood and Built Environment | Estimated Annual Agricultural Pesticide Use - DICAMBA |
| EPEST_HIGH_KG_DIURON | arcsinh_x | Neighborhood and Built Environment | Estimated Annual Agricultural Pesticide Use - DIURON |
| EPEST_HIGH_KG_DODINE | yeojohnson | Neighborhood and Built Environment | Estimated Annual Agricultural Pesticide Use - DODINE |
| EPEST_HIGH_KG_FERBAM | log_x | Neighborhood and Built Environment | Estimated Annual Agricultural Pesticide Use - FERBAM |
| EPEST_HIGH_KG_METHOMYL | arcsinh_x | Neighborhood and Built Environment | Estimated Annual Agricultural Pesticide Use - METHOMYL |
| EPEST_HIGH_KG_METOLACHLOR | boxcox | Neighborhood and Built Environment | Estimated Annual Agricultural Pesticide Use - METOLACHLOR |
| EPEST_HIGH_KG_SETHOXYDIM | arcsinh_x | Neighborhood and Built Environment | Estimated Annual Agricultural Pesticide Use - SETHOXYDIM |
| EPEST_LOW_KG_BROMACIL | yeojohnson | Neighborhood and Built Environment | Estimated Annual Agricultural Pesticide Use - BROMACIL |
| EPEST_LOW_KG_CYHALOTHRIN.LAMBDA | log_x | Neighborhood and Built Environment | Estimated Annual Agricultural Pesticide Use - CYHALOTHRIN.LAMBDA |
| EPEST_LOW_KG_CYPRODINIL | yeojohnson | Neighborhood and Built Environment | Estimated Annual Agricultural Pesticide Use - CYPRODINIL |
| EPEST_LOW_KG_DICAMBA | log_x | Neighborhood and Built Environment | Estimated Annual Agricultural Pesticide Use - DICAMBA |
| EPEST_LOW_KG_DIFLUBENZURON | yeojohnson | Neighborhood and Built Environment | Estimated Annual Agricultural Pesticide Use - DIFLUBENZURON |
| EPEST_LOW_KG_FLUBENDIAMIDE | sqrt_x | Neighborhood and Built Environment | Estimated Annual Agricultural Pesticide Use - FLUBENDIAMIDE |
| EPEST_LOW_KG_FOMESAFEN | sqrt_x | Neighborhood and Built Environment | Estimated Annual Agricultural Pesticide Use - FOMESAFEN |
| EPEST_LOW_KG_IMAZETHAPYR | sqrt_x | Neighborhood and Built Environment | Estimated Annual Agricultural Pesticide Use - IMAZETHAPYR |
| EPEST_LOW_KG_METRIBUZIN | arcsinh_x | Neighborhood and Built Environment | Estimated Annual Agricultural Pesticide Use - METRIBUZIN |
| EPEST_LOW_KG_THIOPHANATE.METHYL | sqrt_x | Neighborhood and Built Environment | Estimated Annual Agricultural Pesticide Use - THIOPHANATE.METHYL |
| MGV_PER_CAPITA_STD_OP | sqrt_x | Health Care Access and Quality | Per capita Medicare outpatient standardized payment |
| MMD_CHRONIC_PQI_F_RATE | log_x | Health Care Access and Quality | Prevention quality chronic composite per 100,000 female Medicare (dual and non-dual) beneficiaries |
| MMD_OUD_IND | exp_x | Health Care Access and Quality | Prevalence of the overarching Opioid Use Disorder (OUD) indicator among Medicare (dual and non-dual) beneficiaries |
| MMD_OVERALL_PQI_OTHER_RATE | yeojohnson | Health Care Access and Quality | Prevention quality overall composite per 100,000 other race Medicare (dual and non-dual) beneficiaries |
| MMD_READM_AIAN_RATE | exp_x | Health Care Access and Quality | All cause readmissions per 100 American Indian or Alaska Native admissions |
| NEPHTN_PCT_ARSENIC_MCL_LESS10 | no_transform | Natural Environment | Percentage of population in the county served by community water systems with yearly distribution of mean arsenic concentration <10 |
| NOAAC_MAX_TEMP_MAY | no_transform | Natural Environment | Monthly (May) maximum temperature (Fahrenheit) |
| NOAAC_PRECIPITATION_FEB | yeojohnson | Natural Environment | Monthly (February) precipitation (Inches) |
| NOAAS_TOT_DROUGHT | exp_x | Natural Environment | Total number of drought |
| NOAAS_TOT_HAIL | log_x | Natural Environment | Total number of hail events |
| NOAAS_TOT_HEAT_EVENTS | center_scale | Natural Environment | Total number of heat events |
| NOAAS_TOT_WIND | log_x | Natural Environment | Total number of wind events |
| PC_PCT_MCARE_MAY_ACPT_APPRVD_AMT | yeojohnson | Health Care Access and Quality | Percentage of clinicians who may accept the Medicare Approved Amounts |
| PC_PCT_MEDICARE_APPRVD_FULL_AMT | exp_x | Health Care Access and Quality | Percentage of clinicians who accept Medicare Approved Amounts in full |
| POS_HOSP_PED_ICU_RATE | no_transform | Health Care Access and Quality | Total number of hospitals with pediatric ICU per 1,000 population |
| POS_MAX_DIST_ED | center_scale | Health Care Access and Quality | Maximum distance in miles to the nearest emergency department, calculated using population weighted tract centroids in the county |
| POS_MIN_DIST_CLINIC | boxcox | Health Care Access and Quality | Minimum distance in miles to the nearest health clinic (FQHC, RHC), calculated using population weighted tract centroids in the county |
| POS_NF_RATE | log_x | Health Care Access and Quality | Total number of nursing facilities per 1,000 population |
| POS_PCT_HOSP_GOV | yeojohnson | Health Care Access and Quality | Percentage of hospitals that are government (federal, state, local, district) hospitals |
| POS_PCT_HOSP_NON_PROFIT | yeojohnson | Health Care Access and Quality | Percentage of hospitals that are private not-for-profit hospitals |
| SC_PVOTE_2014 | exp_x | Social and Community Context | Voter turnout (2nd factor) |
| USCS_INCID_ADJ_RATE | no_transform | Health Care Access and Quality | AGE_ADJUSTED_RATE |

**Table S3.** Temperature variables that were significantly associated with HF readmission and mortality.

| **Exposure** | | **Transformation** | **SD** | **Phase 1** | | | |
| --- | --- | --- | --- | --- | --- | --- | --- |
|  |  |  |  | **Discovery set** | | **Replication set** | |
| **Variable** | **Category** |  |  | **OR (95% CI)** | **q-value** | **OR (95% CI)** | **q-value** |
| Monthly (June) average temperature (Fahrenheit) | Neighborhood and Built Environment | no transform | 0.02 | 1.09 (1.06, 1.12) | 4.73E-06 | 1.06 (1.03, 1.09) | 1.18E-02 |
| Monthly (May) average temperature (Fahrenheit) | Neighborhood and Built Environment | no transform | 0.03 | 1.09 (1.06, 1.12) | 3.03E-06 | 1.06 (1.04, 1.09) | 4.53E-03 |
| Monthly (September) average temperature (Fahrenheit) | Neighborhood and Built Environment | no transform | 0.02 | 1.08 (1.05, 1.11) | 1.20E-04 | 1.06 (1.03, 1.09) | 1.76E-02 |
| Monthly (May) maximum temperature (Fahrenheit) | Neighborhood and Built Environment | no transform | 0.03 | 1.08 (1.05, 1.11) | 3.44E-05 | 1.07 (1.04, 1.1) | 8.13E-04 |
| Monthly (October) maximum temperature (Fahrenheit) | Neighborhood and Built Environment | no transform | 0.04 | 1.09 (1.06, 1.12) | 1.68E-06 | 1.06 (1.03, 1.08) | 3.88E-02 |
| Monthly (June) minimum temperature (Fahrenheit) | Neighborhood and Built Environment | no transform | 0.03 | 1.09 (1.06, 1.12) | 2.87E-06 | 1.06 (1.03, 1.09) | 8.80E-03 |

**Table S4.** STROBE checklist**.**

| **Title and abstract** | | Item No | Recommendation | Page/section |
| --- | --- | --- | --- | --- |
|  | | 1 | (*a*) Indicate the study’s design with a commonly used term in the title or the abstract | Title page, Abstract |
|  |  |  | (*b*) Provide in the abstract an informative and balanced summary of what was done and what was found | Abstract |
| Introduction | | | | |
| Background/rationale | | 2 | Explain the scientific background and rationale for the investigation being reported | Introduction |
| Objectives | | 3 | State specific objectives, including any prespecified hypotheses | Introduction - Study Aim |
| Methods | | | | |
| Study design | | 4 | Present key elements of study design early in the paper | Methods |
| Setting | | 5 | Describe the setting, locations, and relevant dates, including periods of recruitment, exposure, follow-up, and data collection | Methods |
| Participants | | 6 | (*a*) Give the eligibility criteria, and the sources and methods of selection of participants. Describe methods of follow-up | Methods - Data Source and Study population |
|  |  |  | (*b*) For matched studies, give matching criteria and number of exposed and unexposed |  |
| Variables | | 7 | Clearly define all outcomes, exposures, predictors, potential confounders, and effect modifiers. Give diagnostic criteria, if applicable | Methods - Exposures of interest, Methods – Covariates, Table 1 |
| Data sources/ measurement | | 8* | For each variable of interest, give sources of data and details of methods of assessment (measurement). Describe comparability of assessment methods if there is more than one group | Methods - Data Source and Study population, Methods - Statistical Analysis, Table 1 |
| Bias | | 9 | Describe any efforts to address potential sources of bias | Methods - Statistical Analysis |
| Study size | | 10 | Explain how the study size was arrived at | Methods - Data Source and Study population, Figure S2 |
| Quantitative variables | | 11 | Explain how quantitative variables were handled in the analyses. If applicable, describe which groupings were chosen and why | Methods - Statistical Analysis, Figure S1 |
| Statistical methods | | 12 | (*a*) Describe all statistical methods, including those used to control for confounding | Methods - Statistical Analysis |
|  |  |  | (*b*) Describe any methods used to examine subgroups and interactions | Methods - Statistical Analysis, Figure S1 |
|  |  |  | (*c*) Explain how missing data were addressed | Methods - Statistical Analysis |
|  |  |  | (*d*) If applicable, explain how loss to follow-up was addressed | Not applicable |
|  |  |  | (*e*) Describe any sensitivity analyses | Methods - Statistical Analysis |
| Results | | | |  |
| Participants | | 13* | (a) Report numbers of individuals at each stage of study—eg numbers potentially eligible, examined for eligibility, confirmed eligible, included in the study, completing follow-up, and analyzed | Results, Figure S2 |
|  |  |  | (b) Give reasons for non-participation at each stage | Results, Figure S3 |
|  |  |  | (c) Consider the use of a flow diagram | Results, Figure S4 |
| Descriptive data | | 14* | (a) Give characteristics of study participants (eg, demographic, clinical, social) and information on exposures and potential confounders | Results, Table 2 |
|  |  |  | (b) Indicate the number of participants with missing data for each variable of interest | Methods - Statistical Analysis |
|  |  |  | (c) Summarize follow-up time (eg, average and total amount) | Methods - Study Population |
| Outcome data | | 15* | Report numbers of outcome events or summary measures over time | Results |
| Main results | | 16 | (a) Give unadjusted estimates and, if applicable, confounder-adjusted estimates and their precision (eg, 95% confidence interval). Make clear which confounders were adjusted for and why they were included | Results |
|  | |  | (b) Report category boundaries when continuous variables were categorized | Methods - Study outcome, Exposures of interest, Covariates, Area Deprivation Index, Statistical Analysis |
|  | |  | (c) If relevant, consider translating estimates of relative risk into absolute risk for a meaningful time period | Not applicable |
| Other analyses | | 17 | Report other analyses done—eg, analyses of subgroups and interactions, and sensitivity analyses | Results |
| Discussion | | | | |
| Key results | 18 | Summarize key results regarding study objectives | | Discussion - Principal Results, Comparison with Prior Work |
| Limitations | 19 | Discuss limitations of the study, considering sources of potential bias or imprecision. Discuss both the direction and magnitude of any potential bias | | Discussion - Limitations |
| Interpretation | 20 | Give a cautious overall interpretation of results, considering objectives, limitations, multiplicity of analyses, results from similar studies, and other relevant evidence | | Discussion - Conclusions |
| Generalizability | 21 | Discuss the generalizability (external validity) of the study results | | Discussion - Limitations |
| Other information | | | | |
| Funding | 22 | Give the source of funding and the role of the funders for the present study and, if applicable, for the original study on which the present article is based | | Conflicts of Interest |

*Give information separately for exposed and unexposed groups.
