# Supplementary figures and images for: External Exposome Factors and Adverse Heart Failure Outcomes in the OneFlorida+ Network: Retrospective Cohort Study
Source: JMIR Form Res. 2025 Aug 25;9:e71595. doi: 10.2196/71595 (PMC12377874; doi:10.2196/71595)

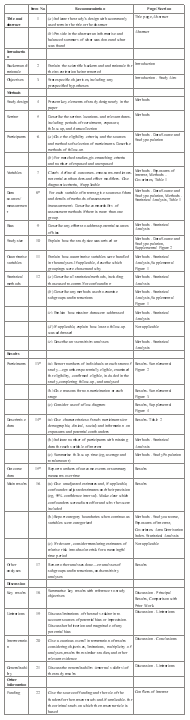

Supplement: Checklist 1 [file formative-v9-e71595-s002.png]
